# Account of a Case of Fistula Lachrymalis; with Reflections on the Different Modes of Operating in That Disease

**Published:** 1808-08

**Authors:** 

**Affiliations:** Baltimore, in America


### Account *of a* Case *of* Fistula Lachrymalis; *with Reflections on the different Modes of operating in that Disease. By Dr.* Chatard, *of Baltimore, in America. In a Letter to Dr.* Miller.

HAVING performed the operation for *Fistula Lachrymalis* about eighteen months ago, I now send you some observations on the subject, which you are at liberty to publish if you find them worthy of consideration.

Nancy ——, of this city, fifteen years of age, for about a year and nine months had been labouring under a *Fistula Lachrymalis* of the worst kind. When first consulted on her case, I found the eye inflamed; the eye-lids red and thickened; a purulent discharge proceeding from an opening situated on the edge of the orbit, about four or five lines below the straight tendon of the orbicularis muscle; the nostril of that side dry, being no longer moistened by the tears which, in a healthy state, incessantly bedew these parts, but which, in this case, were discharged with the pus from the fistulous opening. My first step was to order an emollient poultice, with a view to lessen the inflammation and thickening, which speedily yielded to this application. As soon as it was in my power to examine the condition of the lachrymal passages, I introduced a probe both by the puncta and the fistulous opening; but found it impossible to get beyond the lachrymal sac. Knowing that, in order to relieve this disease, it was necessary to arm myself with patience, and frequently to repeat and vary my attempts, I inserted my probe, and threw injections into the lachrymal ducts every morning and evening for more than two months, without ever being able to reach the nasal duct, where the cause of the disease undoubtedly was seated. Despairing of success, at length, by this method, I proposed the operation, which was performed in the following manner.

The patient being placed in a chair, and her head drawn back and supported on the breast of an assistant, who held it firmly with his hands, I drew down the lower eye-lid with my thumb and fore-finger, so far as perfectly to distinguish the course of the straight tendon of the orbicularis muscle, and the direction of the lachrymal sac. I then pushed the knife into the space below the tendon, and made an oblique incision from above downwards, and from within outwards, of the extent of four lines, in which the fistulous opening was included. Then substituting my probe for the knife, I endeavoured, without success, to pass it into the nasal duct, which seemed to me to be firmly obstructed, as it did likewise to two surgical friends who were present at the operation.

I determined to keep open this small wound for forty-eight hours by means of prepared sponge, with the view of partially destroying the obstacle by the supervening suppuration. At the end of that time the operation was again undertaken, with the intention of pursuing the plan of Hunter, if it should prove impossible to recover the course of the natural passages. But I was very agreeably surprised to perceive by my instrument a removal of that obstruction in the nasal duct which, two days before, had existed in such force. Without withdrawing the bistouri, I immediately introduced, by the direction of it, a kind of curved and flexible probe, which I pushed, without much difficulty, into the cavity of the nose, as appeared by the discharge of some drops of blood from the corresponding nostril. The next object was to bring out of the nostril the lower end of the probe, in which there was a small oval opening for the purpose of introducing some threads of a cord intended to form a seton, which was designed to be enlarged every day by the addition of one or two threads, according to the necessity of dilating the canal more or less, until the obstruction should be perfectly removed. In order to accomplish this, I at first took hold of it with a forceps; but it slipped from the teeth of this intrument without being in the least brought down. It immediately occurred to me to place the groove of a director under the end of the probe, and thus to make it slide below the nostril along the course of the director; which completely succeeded, and answered all the purposes of the appropriate instrument of M. Cabanis, called *palettes*. I then fixed the seton by withdrawing upwards the probe armed with the cord, which was confined firmly along the cheek by a strip of adhesive plaster, which also prevented the access of the air. On the next day, and for several following days, the seton was renewed every morning, without occasioning the least pain to the patient, by means of a thread, tied to the fresh seton, which I took care to draw from below upwards, in order to prevent any depression or sinking of the lips of the wound. At the end of eight days, the swelling being entirely dissipated, and every circumstance proceeding as well as could be wished, I attempted, by passing a probe through the upper lachrymal punctum, to reach the cavity of the nose; but for several days my attempt was fruitless. The end of the instrument was constantly stopped at the orifice of the nasal duct, where I discovered an obstruction formed by a fleshy excrescence, which I removed by a small bistouri with a narrow blade. After this, the communication through the lachrymal ducts, and between the sac and the nasal duct, appeared to be free; and I took care to preserve this freedom, by passing a probe, from time to time, through the upper lachrymal punctum, and by causing to pass into the cavity of the nose a fluid injected through that punctum. I ought, however, to observe, that though it was alyays easy to introduce the probe through the upper lachrymal punctum into the cavity of the nose, the injection often failed to obtain a passage, and wholly regurgitated through the opening in the sac and the lower punctum. I could assign no other reason for this singular fact, than to suppose that the injection, by carrying along in its course a large quantity of purulent matters of different consistence, in some measnre obstructed the passage, and thereby created an impediment to any further progress. However this may be, things proceeded in the manner described for more than three months, and at every dressing the seton was found to be covered with a white and well-conditioned pus in the greater part of its extent; though still some dark specks, now and then, of larger size, were to be seen in it, so as to leave not the least doubt of the existence of a considerable caries in the part. Different injections of appropriate kinds were successively employed to remove this caries; and, at the same time, the remedies adapted to correct the mass of fluids, such as aperients and evacuants, exhibited in a variety of forms, were not neglected.

Finally, after six months of assiduous dressing every morning and evening, I thought it proper to withdraw the seton, which was covered only with the natural secretion of the part; and the ulcer cicatrized without the least deformity in the affected eye, although the lips of this ulcer towards the end of the treatment, were still sufficiently wide to allow the passage of a seton, consisting of twenty-four threads of the ordinary thickness. In the beginning, indeed, I had used one composed of thirty-two threads.

This patient, whose cure has now been effected more than a year, has not since experienced any inconvenience other than a slight obstruction of the tears in the upper lachrymal punctum, where they assume a milky colour, if not wiped once or twice a day, by pressing the finger over the punctum and duct. In all other respects the parts appear in no degree diseased, and it is only by a close inspection that any discovery can be made of an operation having been performed.

### REFLECTIONS.

The Fistula Lachrymalis is a disease by no means rare, and which is too often abandoned to the unsuccessful efforts of nature. Whence does this arise? Apparently, from three sources. 1. The patient is apprehensive on account of the delicacy of the affected organ, and is alarmed, as well at the length of time necessary to accomplish a cure, as at the severity of the means employed by some practitioners in the treatment. 2. Whatever may be the method of treatment, the cure frequently fails, or is left incomplete, without the least deficiency in the assiduities or skilfulness of the surgeon. 3. Professinoal men, who so often find occasion to complain of services ill requited by the public, are unwilling to expose themselves to censure for the trifle of emolument or reputation they can expect to gain from the difficult and doubtful treatment of such cases. Still, however, when we consider the great number of publications on the diseases of the *lachrymal passages*, it seems at first view impossible to resist the belief, that the boundaries of our knowledge on this subject must surely be extended to the *ne plus ultra*. The surgeon, however, who undertakes the task of reading and of operating, will soon perceive that his predecessors have not done every thing for him that might be wished. May I be allowed briefly and explicitly to state my impressions on this subject?

It is clear there are only two modes of giving relief in *fistula lachrymalis*. It is necessary either to restore the natural passages, or to form new ones to supply their place. Among those who adopt the former plan, we may reckon Anel, Mejau, Petit, Monro, Laforet, Palluci, Pouteau, Le Cat, Louis, Heister, Cabanis, M. Desault, and a great many others. Some among these, such as Anel and Laforet, undertake the cure by probing the lachrymal passages, and by throwing injections into them, with more or less frequency, as well by the puncta as by the cavity of the nose. Others combine with this treatment the use of a cord employed as a seton, which they retain for a longer or shorter time in the lachrymal passages, and assert that, by by this means, they are enabled sufficiently to open the obstructed canal. This method was adopted by Mejau. A third party penetrate into the sac by making an incision in the lower eye-lid, nearly similar to that described in the case before related; but afterwards they pursue a different course to open the nasal duct. Petit employed a grooved probe, with which he forced the obstruction; and which, after this difficulty was overcome, served the purpose of introducing a bougie proportioned to the diameter of the opening. Desault employed a cord of catgut, which, in his opinion, was preferable, because it has the quality of swelling, and thereby dilating the duct, in an easy and uniform manner. Other surgeons, after clearing the duct, introduce into it, with a view of its permanently remaining there, a tube of lead, silver or gold, over which they cause the fistula to heal and cicatrize, without perplexing themselves with the unfavourable consequences sometimes resulting from this treatment. My time would not suffice to relate all the numerous varieties of practice recommended by the surgeons who appear as advocates and partizans of the plan of treating this disease by a restoration of the natural passages.

Among those, on the other hand, who have judged it most eligible to form an artificial passage for the tears (and of this number we may mention Voolhouse, St. Ives, Lamorier, Platner, Scabinger, and, above all, the celebrated Hunter), some only undertake to pierce the *os unguis* with the point of a bistouri, and to keep the passage open by means of some foreign body, which they retain there until they judge it might be withdrawn without any risk of the opening closing up; while others break through that bone, nearly in the whole of its extent, in a variety of modes; or, after the example of Hunter, they removed a piece of the bone by a suitable instrument.

As, from the results of these different modes of operating, it appears that all of them have alike succeeded in certain cases, it is natural to infer that none of them are essentially wrong, and that an appropriate selection is the only question of any importance. I conceive, therefore, that where the fistula is recent, the passages only slightly obstructed, and no callosity or caries has taken place, the method of Anel, Laforet, and Mejau, will succeed. In cases of an opposite description it will be necessary to have recourse to the plan recommended by Petit, Desault and others. But when on the contrary, the lachrymal puncta, as well as the common duct, being unaffected, the nasal passage is destroyed by exostosis or otherwise, or the membran which lines it is become entirely callous, or its openin into the nasal cavity is so closed by adhesion as to be ren dered irrecoverable, the treatment recommended by Hun ter will in such cases alone give relief. And if (supposin even a worse case), in addition to the complicated circum stances above stated, there should be an obliteration o the puncta and ducts, even here it seems probable to me that, by means of a double operation, a course might still be procured for the passage of the tears. Dr. Anthony Petit, of Paris, in case of the destruction of the lachrymal puncta, while the nasal duct remains free, proposes to make an artificial passage for the tears between the caruncula lachrymalis and the edge of the lower eye-lid, and to make it in such a manner, that, after obtaining a cicatrix of this small wound, there may permanently remain an opening sufficiently large for a free passage. In such a case as this, would it not be practicable, at the same time, to open a passage into the cavity of the nose, by removing a portion of *os unguis,* according to Hunter's method, in a direction the most oblique, in order, by that means, to increase the slope of the passage for the tears? To obtain this object appears to me not only to be practicable, but to be exceedingly easy.

Although the tumour of the lachrymal sac belongs not to the subject I am treating, since, properly speaking, it is not a fistula; yet as it has been so considered by some authors, I think it proper to observe, that both experience and reason equally prove that it ought to be managed as the simple fistula, and that if the compression so generally recommended to relieve it hath sometimes succeeded, it ought still to be regarded as only a secondary means, and merely adapted to restore to the sac the tone lost by excessive distension. This compression, for instance, would be quite useless in that kind of tumour of the sac observed by M. Petit, the surgeon, which is not diminished by pressure, but which remains hard, and makes constant resistance to the finger. That surgeon supposes this tumour to take place in consequence of violent inflammation, which has had the effect of obstructing, at the same time, the lachrymal puncta and the nasal duct. The tears, then, contained in the lachrymal sac are sufficient to form this tumour, which will remain free of pain as long as they undergo no morbid change; but when they suffer degeneracy, and excite inflammation in the sac, a fistula may then take place, which will be more or less difficult to cure, in proportion portion to the obstacles to be encountered in the restoration of the lachrymal passages, if, indeed, it be practicable to accomplish it. But it is plain that, except the falling of tears over the cheeks, the disease may be relieved; since, for this purpose, it is only necessary to convert this fistula into a simple wound.

In this brief analysis of the different methods adopted by surgeons, I have designedly omitted to give the preference to any one; though I admit that all do not combine the same advantages. The understanding of the surgeon ought to modify his procedure according to the circumstances of the case; for it is a fact, that he will rarely operate twice in precisely the same manner. This was the opinion of the celebrated Desault, whose loss France will long have occasion to lament. And I can declare that, during three years which I passed in studying surgery under that great master, I saw him constantly varying his mode of treatment; so many resources and such facility of accommodation, had he in his creative mind.

After the account now given of the various treatment of this disease, I leave it to every one to make his remarks on that which I adopted. It may be thought that my repeated incision was unnecessary; that the probe with which I opened the nasal duct, and which is nearly like that used by Monro, would be apt to lacerate the sac, and produce dangerous inflammation; that it is difficult to withdraw it from the nostril, and, indeed, impossible in cases where the opening of the duct into the cavity of the nose is diseased in a manner sometimes observed; that the seton I used was too large; and that, by passing it through the wound, I occasioned an irregular and deformed cicatrix. To all this I have but one answer to give: I did what appeared to me to be my duty; on another occasion I shall, perhaps, act conformably to the opinion of the objector, by pursuing my own, and by attending to the indication of the moment. If, in the case above related, I have not restored the organ to its natural state, it is because it was, perhaps, impossible entirely to do so; or that in order to accomplish it, it would have been necessary, after the cure of the fistula, to inject the superior lachrymal duct continually for several months, and to pursue the plan of Mejau in all its extent. But the want of time on my part, the loss of it to the patient, already fatigued by the tediousness of the treatment, and the possibility that nature, occasionally assisted by gentle compression, might complete the rest, determined me to content myself with having freed my pa-

 tient tient from an ulcer, which, to say nothing else, exceedingly disfigured her. And, finally, if by pointing out what I have done, and, perhaps ought to have done, in order to obtain complete success, I can awaken the attention of my younger professional brethren, excite their emulation, and contribute any thing towards enhancing the good they may accomplish in similar cases, I shall flatter myself with the hope of having, in some measure, fulfilled the duty which every practitioner ought to feel incumbent on him, that of being useful to the profession, even although his exertions to be so should be made at the expense of his self-love.

